# Functional opsin retrogene in nocturnal moth

**DOI:** 10.1186/s13100-016-0074-8

**Published:** 2016-10-19

**Authors:** Pengjun Xu, Roberto Feuda, Bin Lu, Haijun Xiao, Robert I. Graham, Kongming Wu

**Affiliations:** 1 State Key Laboratory for Biology of Plant Diseases and Insect Pests, Institute of Plant Protection, Chinese Academy of Agricultural Sciences, No. 2 West Yuan Ming Yuan Road, Beijing, 100193 People’s Republic of China; 2 Tobacco Research Institute, Chinese Academy of Agricultural Sciences, No. 11 Ke Yuan Jing Si Road, Qingdao, 266101 People’s Republic of China; 3 Division of Biology and Biological Engineering, Californian Institute of Technology, Pasadena, CA 91125 USA; 4 Department of Herpetology, Chengdu Institute of Biology, Chinese Academy of Sciences, Chengdu, 610041 People’s Republic of China; 5 Crop and Environment Sciences, Harper Adams University, Edgmond, Shropshire TF10 8NB UK

**Keywords:** Gene duplication, Expression, Evolution, Noctuidae

## Abstract

**Background:**

Retrotransposed genes are different to other types of genes as they originate from a processed mRNA and are then inserted back into the genome. For a long time, the contribution of this mechanism to the origin of new genes, and hence to the evolutionary process, has been questioned as retrogenes usually lose their regulatory sequences upon insertion and generally decay into pseudogenes. In recent years, there is growing evidence, notably in mammals, that retrotransposition is an important process driving the origin of new genes, but the evidence in insects remains largely restricted to a few model species.

**Findings:**

By sequencing the messenger RNA of three developmental stages (first and fifth instar larvae and adults) of the pest *Helicoverpa armigera*, we identified a second, intronless, long-wavelength sensitive opsin (that we called LWS2). We then amplified the partial CDS of LWS2 retrogenes from another six noctuid moths, and investigate the phylogenetic distribution of LWS2 in 15 complete Lepidoptera and 1 Trichoptera genomes. Our results suggests that LWS2 evolved within the noctuid. Furthermore, we found that all the LWS2 opsins have an intact ORF, and have an ω-value (ω = 0.08202) relatively higher compared to their paralog LWS1 (ω = 0.02536), suggesting that LWS2 opsins were under relaxed purifying selection. Finally, the LWS2 shows temporal compartmentalization of expression. LWS2 in *H. armigera* in adult is expressed at a significantly lower level compared to all other opsins in adults; while in the in 1^st^ instar stage larvae, it is expressed at a significantly higher level compared to other opsins.

**Conclusions:**

Together the results of our evolutionary sequence analyses and gene expression data suggest that LWS2 is a functional gene, however, the relatively low level of expression in adults suggests that LWS2 is most likely not involved in mediating the visual process.

**Electronic supplementary material:**

The online version of this article (doi:10.1186/s13100-016-0074-8) contains supplementary material, which is available to authorized users.

## Findings

### Background

Gene duplication is a fundamental process in genome evolution generating new biological functions and promoting adaptation to the changing environment [1–6]. The classical model predicts that after gene duplication, one of the two genes usually degenerates in a few million years, or, in rare cases, one of the duplicates might evolve new gene functions [7]. There are four well established mechanisms by which the DNA duplicates. These are: 1) unequal crossing-over, 2) duplicative (DNA) transposition, 3) polyploidization, and 4) retrotransposition [8]. The contribution of these mechanisms, (with the exception of retrotransposition) to the origin of new genes is well-established. Retrotransposition is different to the other three mechanisms as it includes an intermediate RNA step. That is, a mature mRNA is reverse transcribed into a complementary DNA copy without introns and then inserted back into the genome randomly [9]. The location of insertion is presumed to be random, thereby implying that the copy usually lacks the regulatory apparatus responsible of driving the correct gene expression. This is one of the main reasons why, until recently, retrotransposed genes were not considered to be functional [10–12]. However, recent work has challenged this view suggesting that several retrotransposons have been important for the evolution of novel phenotypes [9]. Interestingly, Marques et al. proposed that between 0.5 and two retrogenes are fixed every million years in flies and mammals, respectively [13–15]. The integration of statistical and molecular methods can be employed to assess the retrotransposons functionality. As reviewed by Kaessman et al. [16] a ratio between non-synonymous and synonymous mutation (ω value) of less than 1 suggests that the gene is under selection and hence still functional. Additionally, other features such as the presence of an open reading frame (ORF), conserved sequence to other species and expression (e.g. qPCR or RNA-seq) are indicative of their functionality.

Opsins are a subfamily of G-protein coupled receptors crucial for the visual process in all the metazoans [17]. In insects, this process is mediated by four paralogs: Long wave sensitive (LWS), Ultraviolet sensitive (UV), Blue sensitive (B) and probably Rh7 [18, 19]. Additionally, it is believed c-opsin might be involved in circadian rhythms [20]. Mechanistically, photoreceptors expressing opsin of different wavelengths signal to the brain, which then perceive colours [21]. The type of wavelength and the separation between maximum peak of absorbance defines the visual capability [18, 19, 22]. Opsin retrotransposons have been identified in various species, including the diurnal moth *Callimorpha dominula* (superfamily Noctuidea) [19], the jellyfish *Tripedalia cystophora* [23], in cephalopods [24], and in teleost fish [25].

In this work, we investigated the evolutionary history of an opsin retrogene in 7 species of nocturnal moth (Noctuidea). Firstly, we sequenced the whole transcriptome for 3 different developmental stages and adults of the pest *Helicoverpa armigera*, including first instar larvae, fifth instar larvae and adults. We identified that in addition to the traditional opsin repertoire (LWS, B and UV), *H. armigera* possesses a second intronless LWS gene (that we call LWS2). Subsequently, we investigated the phylogenetic distribution of this gene in six other Noctuidea species, one Crambidae, 15 Lepidoptera and 1 Trichoptera species. Our results suggest that 1) LWS2 evolved within Noctuidea and 2) the ω-value is less than 1, suggesting a relaxed purifying selection acting on LWS2. Finally, expression levels of LWS2 in *H. armigera* strongly differ between developmental stages, suggesting that most likely this gene is not involved in the visual process.

## Material and methods

### Transcriptome, annotation and opsin identification

Using RNA-Seq, the transcriptome of the whole bodies of first instar larvae (four groups and *n* = 30 for each gourp), fifth instar larvae (four groups and *n* = 10 for each group), and adults (four gourps and *n* = 10 for each group) of the nocturnal moth *H. armigera* was sequenced with paired-end and 100-nt read length on the channels of an Illumina HiSeq™ instrument. Assembled contigs were annotated using BLASTx to align with the database of NR, String, Swissprot and KEGG (see Additional file 1 for details). The RNA-Seq data were submitted to the NCBI GEO database (accession number: GSE86914). Primers were designed and PCR undertaken to (Additional file 1: Table S4) amplify the full-length cDNA of LWS2. To understand whether this duplication was specific to *H. armigera* or species from Noctuidae, we amplified LWS2 in six other species of Noctuidae moth: *Agrotis ypsilon*, *Agrotis segetum*, *Mamestra brassicae*, *Mythimna separata*, *Spodoptera exigua*, *Spodoptera litura* and one species of Crambidae: *Ostrinia nubilalis*. Additionally, we employed BLASTn algorithm to investigated the presence of LWS2 in other 15 complete Lepidoptera and 1 Trichoptera complete genomes from LepBase [26], spanning at total of 12 insect families (see Additional file 1: Table S5). Sequences were identified as follow: LWS1 and 2 were used as seed in a BLASTn search. Each sequences with an e-value <10^−20^ was retained a putative good opsin. To discriminate between opsin and other GPCRs after the translation in protein using TranslatorX [27] and using InterProScan we identified all the sequences with the retinal binding domain.

### Alignment, phylogenetic and evolutionary analysis

The data set including the newly identified LWS2 from Noctuidea, *Callimorpha domicula* opsins, *Ostrinia nubilalis* opsins and putative LWS2 from LepBase (81 sequences in total available in Additional file 2) was aligned using the codon model as implemented in PRANK [28]. The resulting alignment was manually curated to remove gap-rich regions. GTR-G was identified as the best-fitting substitution-model accordingly to the AIC as implemented in Modelgenerator [29]. The phylogenetic reconstruction was performed using Maximum likelihood (ML) using Iqtree [30] and confirmed using Bayesian analysis (BA) under site heterogeneous model CAT-G model as implemented Phylobayes 3.3e [31]. In ML reconstruction the nodal support was evaluated using UltraFast bootstrap (BS) and abayes (aBS) [32] while in BA using the Bayesian posterior probability (PP). In the BA the convergence among chains was estimated using bpcomp and chains were considered converged when the maxdiff value was < 0.3. The phylogenetic analyses were performed using a rooted tree i.e. the LWS tree was rooted using Rh7, UV and Blue opsin. Finally, in order to account for the possible misleading effect of using distant related outgroup, we repeated the analyses without outgroup.

In order to estimate whether LWS2 is under evolutionary constraint we estimated the ratio between non-synonymous and synonymous substitutions (ω-value) using a maximum likelihood approach [33] as implemented in CODEML [34]. If the ω < 1, this is indicative of purifying selection However, if the retrogenes were under relaxed purifying selection, we expect an elevated ω-value relative to its paralog LWS1, which suggests that it may be neo-functionality or sub-functionalization. We evaluated five hypotheses: (1) one ω-ratio for all branches (one-ratio model; assuming that all branches have been evolving at the same rate); (2) ω-ratio = 1 for all branches (neutral model; neutral evolution for all branches); (3) moth LWS2 lineage and LWS1 lineage have different ω-ratio (ω_2_ and ω_1_; two ratio model; allowing foreground branch to evolve under a different rate); (4) neutral evolution for moth LWS2 lineage (ω_2_ = 1); and (5) the free-ratio model with free ω-ratio for each branch. In addition, we used branch-site models: moth LWS2 lineage was defined as the foreground, rest lineages were defined as the background branch, and these were then specified in the tree file by using branch labels. Likelihood-ratio test (LRT) was employed to determine if the alternative model, indicating positive selection, was superior to the null model. We also performed CODEML test on *H. armigera* LWS2 lineage to see if nature selection acted on any of the LWS2 branches.

### Quantitative expression of opsin

To test the expression of the opsin in *H. armigera*, we investigated the relative level of expression in the 3 different developmental stages using fragments per kilobase of exon per million fragments mapped (FPKM) [35]. Differential gene expression between paralogs at different developmental stages was evaluated using STATA v.9.0 and ANOVA. Bonferroni multiple comparisons were used to determine the level of significance between the relative levels of mRNA expression.

## Results and discussion

**Fig. 1:**
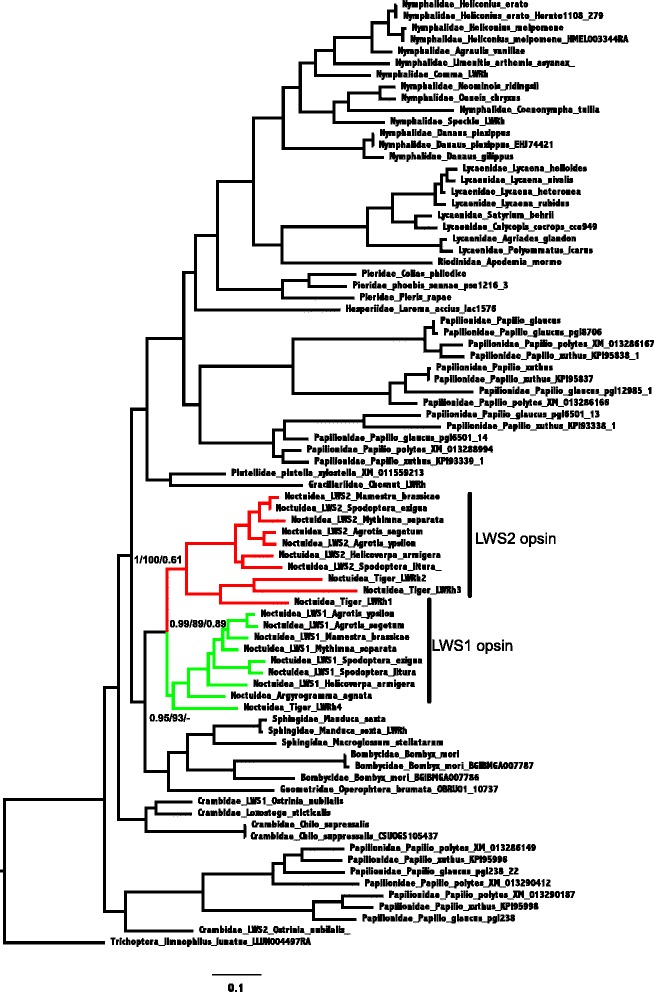
Maximum likelihood under GTR-G. Support values at nodes are from left to right aBayes, Ultrafast bootstrap and Bayesian Posterior Probability. LWS2 opsins of noctuid species denoted in red color. Noctuidae LWS1 opsins denoted in green color

We obtained about 4 gigabase (Gb) of sequence each sample from the RNA-Seq (total 63 Gb for 12 samples), and a total of 99,711 contigs (See Additional file 1 for details; with a total of 73, 709 unigenes). Using functional annotation, we were able to identify that in addition to the traditional insects opsin genes of LWS, UV and Blue [19, 36]. *H. armigera* possesses an additional opsin gene, named LWS2. Using PCR we confirmed the presence of this gene in *H. armigera* and in 6 other Noctuidea species. The phylogenetic analysis displayed in Fig. 1 supports the paralog relationship between the newly identified opsin and LWS2 from other species and LWS1 (PP = 0.76, BS = 1, aBS = 100, see Fig. 1). Additionally, our phylogenetic trees suggests that LWS2 orthologs are present only in Noctuidea. The analysis of the intron content suggests that while LWS1s noctuid species have seven introns, no introns are present in the LWS2s (Additional file 1: Figures S2a and S2b), this finding together with the monophyly of LWS1 and 2 suggests that the later (i.e. LWS2) originated as a retrocopy from LWS1 [8, 16]. Furthermore, our results strongly support the monophyly of intronless opsin in Noctuidea (PP = 0.9, BS = 100, aBS = 1) (Fig. 1). The tree topologies are invariant in respect to the sequences used for rooting the tree (compared to Additional file 1: Figures S3, S4, S5 and S6).

In the next section, we investigated whether these LWS2 are functional. First we observed that all the LWS2 genes identified in this study have intact ORFs (see Additional file 1: Figure S2) arguing in favor of LWS2 functionality. Furthermore, our results suggest that LWS2 have an ω-value <1 (ω = 0.08202) indicating that is evolving under purifying selection as expected in functional genes. Finally we did not detect signal of positive selection acting on LWS2 (Table 1). These findings together indicate that LWS2 it is a functional genes.

**Table 1:** Selective patterns for LWS opsins

Model	np^a^	Ln L^b^	Estimates of ω	Models compared	LRT^c^	*P* Values
Branch model						
A: one ratio	175	−41372.52	ω = 0.06527			
B: one ratio ω = 1	174	−47529.36	ω = 1	B vs. A	12313.68	0.0
C: the LWS2 branch has ω_2_, the LWS1 branch has ω_1_	177	−41347.92	ω_2_ = 0.08202	A vs. C	49.2	0.0
			ω_1_ = 0.02536			
D: the LWS2 branch has ω_2_ = 1	176	−41783.48	ω_2_ = 1	D vs. C	871.12	0.0
			ω_1_ = 0.02515			
E: each branch has its own ω	347	−41045.37	Variable ω by branch	A vs. E	654.3	0.0
Branch-site models						
G: the LWS2 branch	178	−41152.48				
H: the LWS2 branch has ω = 1	177	−41152.48		H vs. G	0.0	1.0

^a^Number of parameters

^b^The natural logarithm of the likelihood value

^c^Twice the log likelihood difference between the two models

**Fig. 2:**
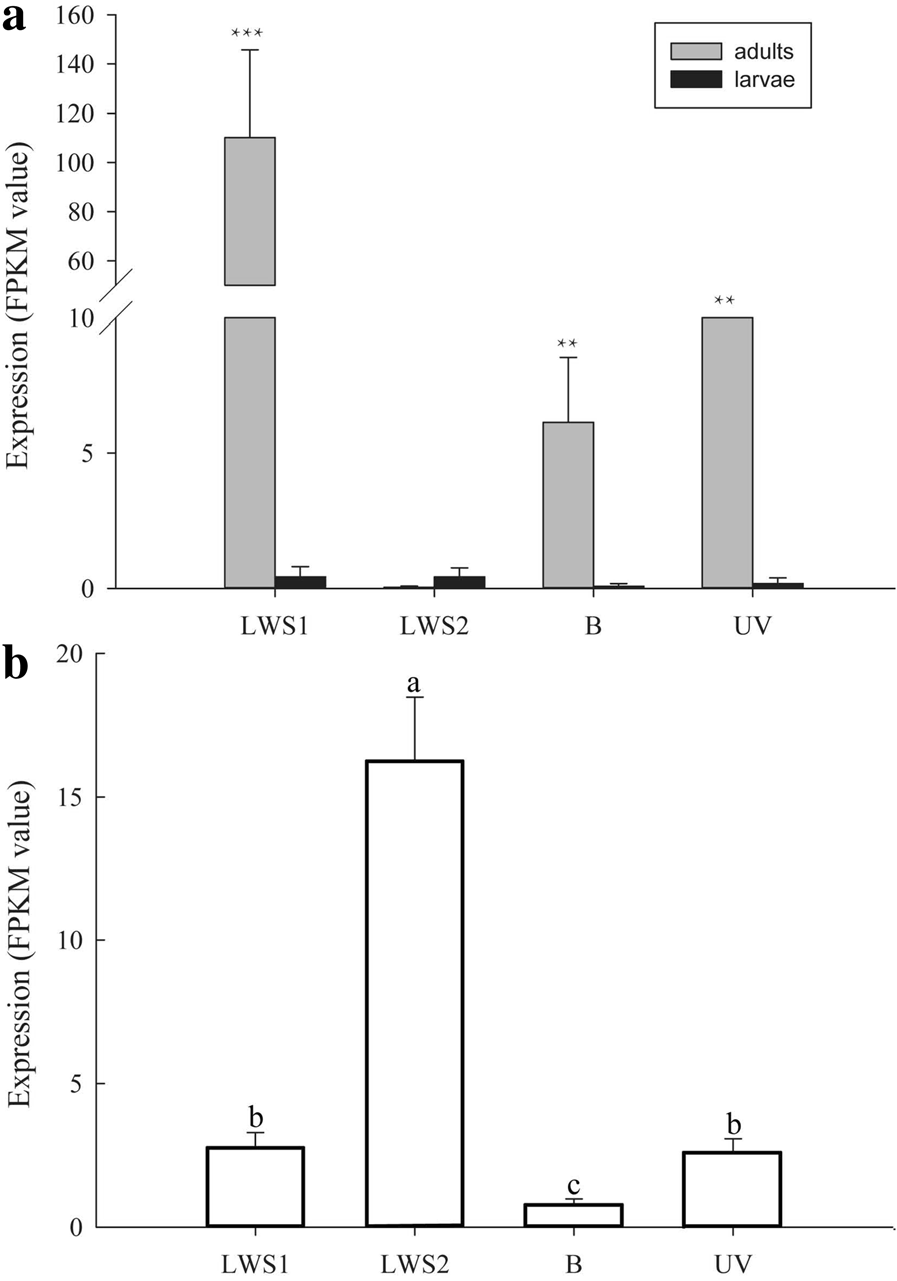
Quantitative expression levels for opsin genes in **a** larvae at 5^th^ instar stage and adults and **b** larvae at 1^st^ instar stage. **a** The “**” denote statistical significance of the expression levels at *P* < 0.01. The “***” denote statistical significance of the expression levels at *P* < 0.001. **b** Significant difference is shown using different letters (*P* < 0.0001, d.f. = 3, 12, F = 203.83). Mean ± SD. *n* = 4

Subsequently, we investigated whether the LWS2 might contribute to the evolution of visual capability in Noctuidea. The data from RNA-seq expression level in *H. armigera* suggested that LWS1, and more generally UV and Blue opsins in *H. armigera*, were significantly higher expressed in adults than LWS2 (Fig. 2a). The result suggested that LWS2 migth be not involved in the visual system of the adult. However, surprisingly, LWS2 in 1^st^-instar larvae has an higher relative level of expression compared to the other three opsins (Fig. 2b). The reasons for higher level of expression of LWS2 at the 1^st^-instar larvae are unclear. This finding is conceivable with a function of LWS2 other than vision [37, 38], another alternative is that observed level of expression represents transcriptional noise. The retrotransposed opsins are expressed as results of transcriptional activity in the new genomic location. However, additional experiments would be necessary to clarify between the competing hypothesis.

In conclusion we report the existence of LWS2, originating as retrocopies from LWS1, in seven moths from the superfamily Noctuoidea. Furthermore, the intact ORF, the ω < 1, the phylogenetic conservation and expression independently suggests that LWS2 opsins are functional.

## Additional files

Additional file 1: Materials and methods Detailed description of materials and methods used in RNA-seq and PCR amplification. **Results** Description of the results in RNA-seq and PCR amplification. **Table S1.** Summary of the sequence assembly after Illumina sequencing. **Table S2.** The opsin genes from *H. armigera* by RNA-seq. **Table S3.** The FKPM values of opsin genes in *H. armgiera*. **Table S4.** Primers used in this study. **Table S5.** The species with complete genome sequence used in this study. **Figure S1.** The discription of RNA-seq. (a) The distribution of sequences length. (b) The E-value distribution of the top matches in the nr database. (c) The species distribution of the matches in the nr database. (d) The sequence similarity distribution. **Figure S2.** The genomic sequence of LWS opsins in seven noctuid species. (a) LWS1 opsins showed seven introns. (b) The fragments od LWS2 opsins from seven noctuid species showed no introns in the region. The red letters showed the homology region of primers for amplifying partial sequence of LWS2 using DNA as templete. The introns are shaded. “.” = identical nucleotides; “-” = absence of nucleotides. AS = *Agrotis segetum*, AY = *Agrotis ypsilon*, HA = *Helicoverpa armigera*, MB = *Mamestra brassicae*, MS = *Mythimna separata*, SELWS1 = *Spodoptera exigua*, SL = *Spodoptera litura*. (c) The genomic sequence of LWS1 in *O. nubilalis*. (d) The genomic sequence of LWS2 in *O. nubilalis*. The exons were showed using black letters and the introns were showed using red letters. **Figure S3.** Maximum Likelihood tree with outgroup. **Figure S4.** Bayesian tree with outgroup. **Figure S5.** Maximum Likelihood tree with outgroup. **Figure S6.** Bayesian tree with outgroup. (DOC 2650 kb)
Additional file 2: Alignment of sequences used in this study. (PHY 100 kb)

## Abbreviations

aBS: a Bayes support
B: Blue sensitive opsin
BI: Bayesian inference
BS: Bootstrap support
FPKM: Fragments per kilobase of exon per million fragments mapped
LRT: Likelihood-ratio test
LWS: Long-wavelength sensitive opsin
ORF: Open reading frame
PP: Posterior probability
UV: Ultraviolet sensitive opsin
