# Supplementary material for: Functional opsin retrogene in nocturnal moth
Source: Mob DNA. 2016 Oct 19;7:18. doi: 10.1186/s13100-016-0074-8 (PMC5070202; doi:10.1186/s13100-016-0074-8)
Supplement: Additional file 1: — Materials and methods Detailed description of materials and methods used in RNA-seq and PCR amplification. Results Description of the results in RNA-seq and PCR amplification. Table S1. Summary of the sequence assembly after Illumina sequencing. Table S2. The opsin genes from H. armigera by RNA-seq. Table S3. The FKPM values of opsin genes in H. armgiera. Table S4. Primers used in this study. Table S5. The species with complete genome sequence used in this study. Figure S1. The discription of RNA-seq. (a) The distribution of sequences length. (b) The E-value distribution of the top matches in the nr database. (c) The species distribution of the matches in the nr database. (d) The sequence similarity distribution. Figure S2. The genomic sequence of LWS opsins in seven noctuid species. (a) LWS1 opsins showed seven introns. (b) The fragments od LWS2 opsins from seven noctuid species showed no introns in the region. The red letters showed the homology region of primers for amplifying partial sequence of LWS2 using DNA as templete. The introns are shaded. “.” = identical nucleotides; “-” = absence of nucleotides. AS = Agrotis segetum, AY = Agrotis ypsilon, HA = Helicoverpa armigera, MB = Mamestra brassicae, MS = Mythimna separata, SELWS1 = Spodoptera exigua, SL = Spodoptera litura. (c) The genomic sequence of LWS1 in O. nubilalis. (d) The genomic sequence of LWS2 in O. nubilalis. The exons were showed using black letters and the introns were showed using red letters. Figure S3. Maximum Likelihood tree with outgroup. Figure S4. Bayesian tree with outgroup. Figure S5. Maximum Likelihood tree with outgroup. Figure S6. Bayesian tree with outgroup. (DOC 2650 kb) [file 13100_2016_74_MOESM1_ESM.doc]

**Additional file 1**

**Material and Methods**

***Insects***

*Helicoverpa armigera*, *Agrotis ypsilon* and *Ostrinia nubilalis* were reared using artificial diet at 25±1 °C with a 14:10, light:dark photoperiod. Adult moths were provided with 10% sugar and 2% vitamin complex. The adults of other five species, including *Agrotis segetum*, *Mamestra brassicae*, *Mythimna separata*, *Spodoptera exigua* and *Spodoptera litura*, were captured and stored in liquid N_2_ in July 2011 using a vertical pointing trap set up on Beihuang Island, Shandong province (38° 23.200’ N, 120° 54,500’ E).

***cDNA library construction and Illumina sequencing for transcriptome analysis***

Four groups of 1^st^ instar larvae on the third day after hatching (30 individuals each group), four group of 5^th^ instar stage larvae on the first day after ecdysis (n=10 for each group) and four group adults (n=10 for each group) on the first day after eclosion of *H. armigera* were used to construct the cDNA library of *H. armigera* using an mRNA-Seq assay for paired-end transcriptome sequencing, which was performed by Novogene (Beijing, China). Poly(A) mRNA was isolated from 20 µl total RNA using Oligo (dT) magnetic beads and then was broken into short fragments (about 200bp) in the presence of fragmentation buffer at 94 °C for 5 min. These short fragments were used as templates for first-strand cDNA synthesis using random hexamer-primers. Subsequently, second-strand cDNAs were synthesized using buffer, dNTPs, RNaseH, and DNA polymerase I. After purification of short fragments with a QiaQuick PCR Purification Kit (Qiagen), samples were then washed with EB buffer for end reparation and single nucleotide adenine addition. Finally, the short fragments were connected to sequencing adapters. Suitable fragments, as judged by agarose gel electrophoresis, were enriched with PCR amplification to prepare the sequencing library. The cDNA library was sequenced on the channels of an Illumina HiSeq™ 2000 instrument for about 4 gigabase in-depth.

***De novo assembly of sequencing reads and assembly***

5’ and 3’ low-quality ends of raw reads were trimmed using Fastx-tools. Adaptor sequences were also trimmed using Fastx-tools. Reads with over 5 Ns or the length of which is shorter than 80bp were removed. The remained pair-end reads were kept as clean reads, which were used for *de novo* assembly. Then, the Trinity (v2.0.6) [1] software were used to assemble the clean reads with default parameters. All assemblies were performed on a server with 48 cores and 128 GB of memory. After assembly, the contigs longer than 200 bases were used for subsequent analysis.

***Quantitation of transcript sequences***

The reads from 8 libraries were mapped to the assembled contigs using Bowtie 0.12.7 [2] with no more than 2 mismatches within the ﬁrst 28 bp. The read counts accumulated on the contigs were normalized as fragments per kilobase of exon model per million mappedreads (FPKM) values [3]. Quantitative analysis for each contig was estimated using FPKM values by RSEM (v1.1.17) software [4] with default parameters.

***Functional annotation***

BLASTx was performed to align the assembled contigs from Trinity to the database of NR, String, Swissprot and KEGG for functional annotation. The e-value cut-off was set at 1E-5 for further analysis. Each assembled contig was assigned with the gene name and related function based on the best BLASTx hit (the smallest e-value). Assembled contigs assigned to the same gene were further compared, and the contig from the best e-value was adopted. If there was a tie between 2 assembled sequences, the one with the largest sequence identity was selected.

***Opsin identification***

Total RNA was isolated from individual adult moths using TRIzol reagent (Invitrogen, Carlsbad, CA, USA). Single stranded cDNA was synthesized using oligo(dT) and M-MLV Reverse Transcriptase (Promega, Madison, WL, USA). Genomic DNA was extracted from each individual using Easy Pure Genomic DNA Extraction Kit (TransGen, Beijing, China). With specific primers designed according to the sequence from RNA-seq and degenerate primers according to the conserved 5’untranslated region (UTR) [5], we amplified the full-length cDNA of LWS2 in *H. armigera* using cDNA as template with a PCR program: 4 min at 94 °C; 30 s at 94 ^o^C, 30 s at 50^o^C, and 60 s at 72 ^o^C for 45 cycles. According to the completed CDS obtained in this study and reported previously [5], specific primers were designed to amplify the genomic sequence of the LWS1 from the seven species and the LWS2 from *H. armigera* and the PCR program: 4 min at 94 °C; 30 s at 94 ^o^C, 30 s at 53^o^C, and 2 min at 72 ^o^C for 45 cycles. Degenerate primers were used to amplify the partial CDS from the other six species using genomic DNA as template with a PCR program: 4 min at 94 °C; 30 s at 94 ^o^C, 30 s at 50^o^C, and 30 s at 72 ^o^C for 45 cycles. All the primers used in this study were shown in Additional file 1. According to the RNA-seq data of *Ostrinia nubilalis* from Dr. Tiantao Zhang (Institute of Plant Protection, Chinese Academy of Agricultural Sciences, Beijing, P.R. China), we designed specific primers to amplify the LWS1 and LWS2 of *O. nubilalis* using cDNA and DNA templates with a PCR program: 4 min at 94 °C; 30 s at 94 ^o^C, 30 s at 55^o^C, and 2 min at 72 ^o^C for 45 cycles.

Statistical analyses were conducted using STATA v.9.0. Student’s t-test or ANOVA with Bonferroni multiple comparisons were used to determine the level of significance in the relative levels of mRNA expression.

**Results**

#### *Illumina sequencing and reads assembly*

After trimming adaptor sequences and removing low-quality reads, a total of about 300 million clean sequencing reads with an average length of 100 bp were generated. An overview of the sequencing and assembly is outlined in table S[1](http://www.biomedcentral.com/1471-2164/14/411/table/T1). The mean contig size was 682 bp, with lengths ranging from 200 to 19580 bp.

#### *Annotation of predicted proteins*

For annotation, BLASTx was used to compare unigenes against the NCBI nr database by using a cut-off E-value of 10^-5^. Using this approach, 47598 genes (39.3% of transcripts) were able to be get BLAST hits using the E-value cutoff. Because of the relatively short length of transcripts (mean size of 628 bp) and lack of genome reference, most of the assembled sequences could not be matched to any known genes. Figure S[1](http://www.biomedcentral.com/1471-2164/14/434/figure/F1)a shows that the proportion of assembled sequences with matches in the nr database is higher among the longer sequences. Specifically, sequences longer than 2000 bp had a match efficiency of 99.0%, whereas the match efficiency decreased to approximately 60.8% for sequences ranging from 500 to 1000 bp in length, and to 38.2% for sequences between 200 and 500 bp in length (Figure S[1](http://www.biomedcentral.com/1471-2164/14/434/figure/F1)a). The E-value distribution of the best hit in the nr database showed that 50% of the mapped sequences have strong homology (smaller than 1.0E-50), whereas 50% of the homologous sequences ranged from 1.0E-5 to 1.0E-50 (Figure S[1](http://www.biomedcentral.com/1471-2164/14/434/figure/F1)b). The sequence similarity distribution has a comparable pattern, with 31% of the sequences having a similarity higher than 80%, and 69% of the sequences having a similarity ranging from 20% to 80% (Figure S[1](http://www.biomedcentral.com/1471-2164/14/434/figure/F1)c, S1d).

***Opsin genes of H. armigera***

According to functional annotation, we found four opsin genes from the assembled contigs (table S2). The fragments per kilobase of exon per million fragments mapped (FPKM) values of each opsin were used to count and analyze the expression profile (table S3).

***Cloning the LWS2 opsins***

Using RNA-seq, we were able to successfully identify partial sequence of the second LWS opsinat 3’end in *H. armigera*, which was used to design specific primer. Taken together with 5’ degenerate primer, we successfully obtained the complete coding domain sequence (CDS) of the LWS2 in *H. armigera* (GenBank accession no.: KJ010188). According to the complete CDS of LWS1 from the seven noctuid species, we designed primers to amplified the genomic sequences of those opsins using genomic DNA as templete. The results indicated that there were seven introns in the LWS1 (Figure S2a). According to the complete CDS of LWS2 from *H. armigera*, we designed primers to amplified the genomic sequences of the opsin using genomic DNA as templete, which was consistent with the CDS, indicating there was no intron in LWS2 of *H. armigera*. According to the sequences of LWS1/LWS2 and the location of introns, we designed primers to amplified the fragments of LWS2 from the six species and the results indicated that there were no introns in the LWS2 of these six species (Figure S2b). According to reference sequences from RNA-seq data, we also amplified the LWS1 and LWS2 in *O. nubilalis* using cDNA and DNA as templete. The results indicated that both the LWS1 and LWS2 of *O. nubilalis* contained introns (Figure S2c, S2d), suggesting a genomic duplication.

**References**

1. Grabherr MG, Haas BJ, Yassour M, Levin JZ, Thompson DA, Amit I, Adiconis X, Fan L, Raychowdhury R, Zeng Q, Chen Z, Mauceli E, Hacohen N, Gnirke A, Rhind N, di Palma F, Birren BW, Nusbaum C, Lindblad-Toh K, Friedman N, Regev A. Full-length transcriptome assembly from RNA-seq data without a reference genome. Nat Biotechnol. 2011;9: 644-652.
2. Langmead B, Trapnell C, Pop M, Salzberg SL. Ultrafast and memory-efficient alignment of short DNA sequences to the human genome. Genome Biol. 2009;10:R25.
3. Trapnell C, Williams BA, Pertea G, Mortazavi A, Kwan G, van Baren MJ, Salzberg SL, Wold BJ, Pachter L. Transcript assembly and quantification by RNA-Seq reveals unannotated transcripts and isoform switching during cell differentiation. Nat Biotechnol. 2010;28: 511-515.
4. Li B, Dewey CN. RSEM: accurate transcript quantification from RNA-Seq data with or without a reference genome. BMC Bioinformatics. 2011;12:323
5. Xu P, Lu B, Xiao H, Fu X, Murphy RW, Wu K. The evolution and expression of the moth visual opsin family. PLoS One. 2013;8:e78140

| The names of samples | L1-1 | L1-2 | L1-3 | L1-4 | L5-1 | L5-2 | L5-3 | L5-4 | AF1 | AF2 | AM1 | AM2 |
| --- | --- | --- | --- | --- | --- | --- | --- | --- | --- | --- | --- | --- |
| Total reads | 7.5G | 5.7G | 5.7G | 6.8G | 4.9G | 4.6G | 4.0G | 5.0G | 5.2G | 4.7G | 5.3G | 3.8G |
| Total number of contigs | 99711 | | | | | | | | | | | |
| Total number of unigenes | 73709 | | | | | | | | | | | |
| N50 of assembled contigs (bp) | 2254 | | | | | | | | | | | |
| Mean length of contigs (bp) | 1018 | | | | | | | | | | | |

**table S1** Summary of the sequence assembly after Illumina sequencing. A=adult, L1=Larve at first instar stage, L5=Larve at fifth instar stage, F=Female, M=Male.

| The names of opsins | Unigene | Length | NR_Hit-Name | NR_E-Value | NR_Similarity | NR_Description |  |  |
| --- | --- | --- | --- | --- | --- | --- | --- | --- |
| LWS1 | c52406_g1 | 1800 | gi|465828050|gb|AGH28027.1| | 0.00E+00 | 100% | long wavelength-sensitive opsin [*Helicoverpa armigera*] | | |
| LWS2 | c57977_g1 | 1572 | gi|557955907|gb|AHA48189.1| | 0.00E+00 | 95% | long-wavelength opsin [*Mamestra brassicae*] | | |
| B | c59975_g1 | 2435 | gi|465828095|gb|AGH28029.1| | 0.00E+00 | 100% | blue wavelength-sensitive opsin [*Helicoverpa armigera*] | | |
| UV | c52984_g2 | 754 | gi|320130496|gb|ADW20311.1| | 0.00E+00 | 100% | ultraviolet wavelength-sensitive opsin [*Helicoverpa armigera*] | | |

**table S2** The opsin genes from *H. armigera* by RNA-seq.

| The names of opsins | L1-1 | L1-2 | L1-3 | L1-4 | L5-1 | L5-2 | L5-3 | L5-4 | AF1 | AF2 | AM1 | AM2 |
| --- | --- | --- | --- | --- | --- | --- | --- | --- | --- | --- | --- | --- |
| LWS1 | 3.145 | 3.155 | 2.589 | 1.9 | 0.47 | 0.242 | 0.939 | 0.064 | 119.674 | 60.657 | 145.223 | 115.271 |
| LWS2 | 17.275 | 18.896 | 14.312 | 14.488 | 0.674 | 0 | 0.328 | 0.714 | 0.045 | 0 | 0.097 | 0.047 |
| B | 0.582 | 0.996 | 0.625 | 0.914 | 0.025 | 0.04 | 0.226 | 0.043 | 4.817 | 3.52 | 8.827 | 7.385 |
| UV | 2.204 | 3.131 | 2.849 | 2.138 | 0 | 0 | 0.283 | 0.426 | 18.794 | 13.947 | 31.568 | 29.152 |

**table S3**The FPKM values of opsin genes in *H. armgiera*. A=adult, L1=Larve at first instar stage, L5=Larve at fifth instar stage, F=Female, M=Male.

| Primer names | Primer sequence (5’-3’) | Amplified fragments |
| --- | --- | --- |
| HaLWS2-F | AAGACTGGAGTAGTTCGTKAARCG | Primers for the full-length cDNA of HaLWS2 |
| HaLWS2-R | GTCGCGACAGAGCCAC |
| LWS2-F1 | AAGACTGGAGTAGTTCG | Degenerate primer for 5’ ends of LWS2 |
| LWS2-LWS1 | TTVGCYTGYTCCCTCATAG |
| ASLWS1-GF | ATGTCGCTGACTCTGGATC | Primers for amplifying genomic sequence of the LWS1 opsin from *Agrotis segetum* |
| ASLWS1-GR | TCAGGCGGCAGGCTTC |
| AYLWS1-GF | ATGTCGCTGA CTCTGGATC | Primers for amplifying genomic sequence of the LWS1 opsin from *Agrotis ypsilon* |
| AYLWS1-GR | TCAGGCGGCAGGTTTC |
| HALWS1-GF | ATGCCGCTGACTCTGGAT | Primers for amplifying genomic sequence of the LWS1 opsin from *Helicoverpa armigera* |
| HALWS1-GR | CTAGGCGGCAGGCTC |
| MSLWS1-GF | ATGTCTCTGAGTCTGGATCC | Primers for amplifying genomic sequence of the LWS1 opsin from *Mythimna separata* |
| MSLWS1-GR | GGCTTCTCCTCGGAGAC |
| MBLWS1-GF | ATGTCGCTGACTCTGGATC | Primers for amplifying genomic sequence of the LWS1 opsin from *Mamestra brassicae* |
| MBLWS1-GR | GGCTTCTCCTCGGAAAC |
| SELWS1-GF | ATGTCGCTGACTTTGGATC | Primers for amplifying genomic sequence of the LWS1 opsin from *Spodoptera exigua* |
| SELWS1-GR | TTAGGCAGCAGGTTTCTC |
| SLLWS1-GF | ATGTCGCTGACTTTGGATC | Primers for amplifying genomic sequence of the LWS1 opsin from *Spodoptera litura* |
| SLLWS1-GR | TTAGGCCGCAGGTTTCTC |
| LW2F-G | ATGGTCATCTACATATTYTCA | Degenerate primers for amplifying partial sequence of the LWS2 opsins using DNA as templete |
| LW2R-G | GATTCCRAGTATGCGCT |
| OnLWS1F | ATGGCAATTGCCAGCTTG | Primers for amplifying g the LWS1 opsin from *Ostrinia nubilalis* using cDNA and DNA as templates |
| OnLWS1R | GCAGCGGGTTTCTCATCG |
| OnLWS2F | ATGGCTACAGGACCAGGAGT | Primers for amplifying g the LWS2 opsin from *Ostrinia nubilalis* using cDNA and DNA as templates |
|  |  |
| OnLWS2R | CAACCTTGGCAAGTTTGC |

**table S4.** Primers used in this study.

**table S5.** The species with complete genome sequence used in this study.

| Names of speceis |
| --- |
| *Bombyx mori* |
| *Calycopis cecrops* |
| *Chilo suppressalis* |
| *Danaus plexippus* |
| *Heliconius erato* |
| *Heliconius melpomene* |
| *Lerema accius* |
| *Limnephilus lunatus* |
| *Manduca sexta* |
| *Melitaea cinxia* |
| *Operophtera brumata* |
| *Papilio glaucus* |
| *Papilio polytes* |
| *Papilio xuthus* |
| *Phoebis sennae* |
| *Plutella xylostella* |


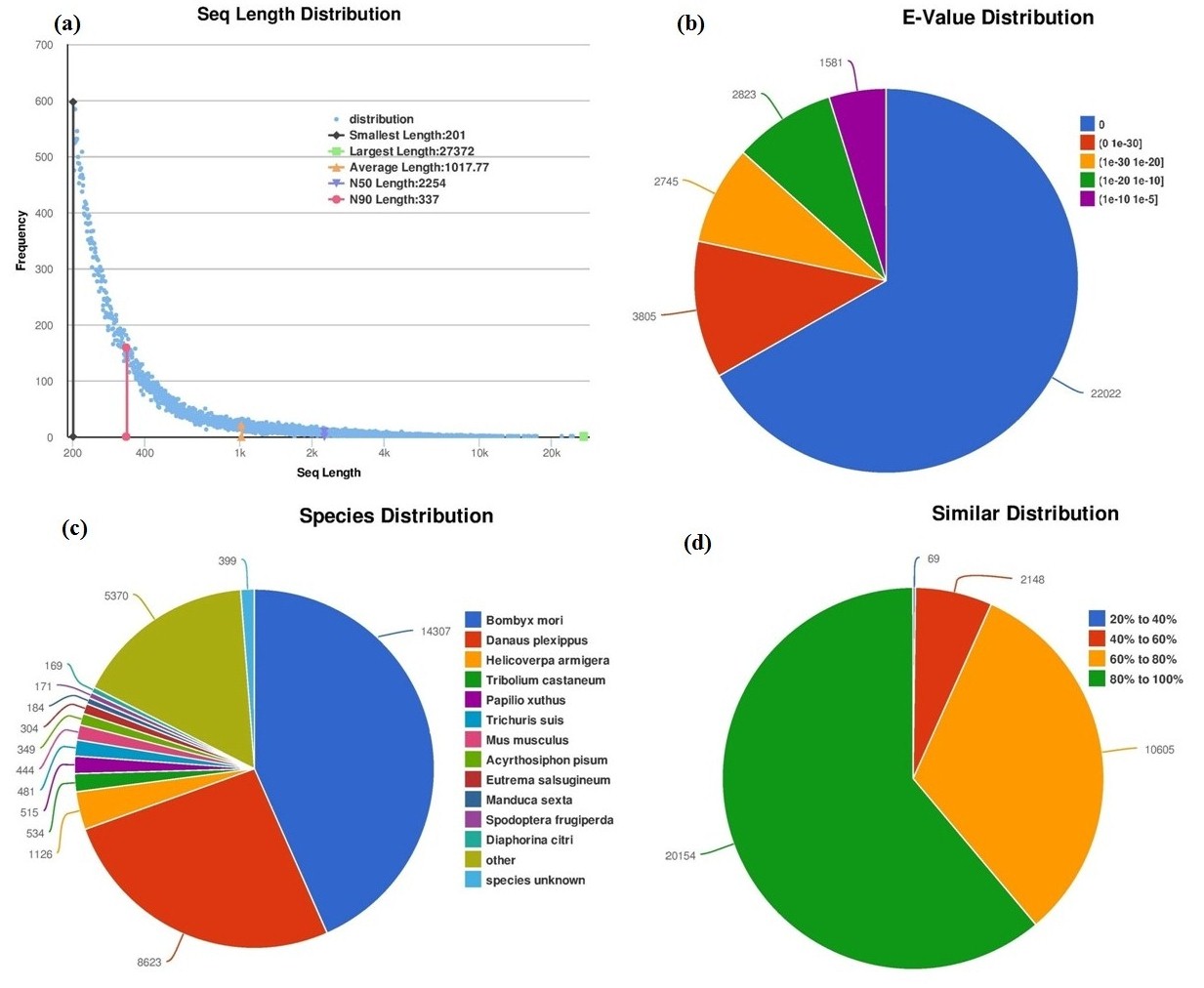


**Figure S**[**1**](http://www.biomedcentral.com/1471-2164/14/434/figure/F1) **The description of RNA-seq.** (a) The distribution of sequences length. (b) The E-value distribution of the top matches in the nr database. (c) The species distribution of the matches in the nr database. (d) The sequence similarity distribution.

**ASLWS1TGGCCAAGTAGCGGCGTATGGCGCCGCCAACCAAACCGTCGTGGACAAGGTGCCGCCAGATATGCTCCATATGGTCGATCCTTACTGGTGAGTCTAAATATTTTTTGAGTTCGTGTTAATTCGTGACCCTCATTATAATA** 140

**AYLWS1.........................T.......G.....G..................................................C....G.......G.......T.....T................T.....** 140

**HALWS1C.....G........A.........T.A.................T........A..C........T..C............C..........G....ACA..A..-------AAC...CATAACTGTG.A.CA.A...T** 133

**MbLWS1......G..................T.......G.....A.................T........T...............C.........GAGTC.GT..AAAATCTA.T...AA..CCTTCA.TTG.TTA..GC..T** 140

**MSLWS1C.....G..................T.......G.......................T..C.....T...............C......C..---TC.AT..AC.CATCA.TA..AAT.C.T.CCTT....CACC.TT.T** 137

**SELWS1............A.....C..T...T................T.....A....TA...........T...............C............G.T.C...GA.TGTCA..GACAG..CTAGCTGAG.AT.A.C..A.** 140

**SLLWS1.....................T...T................T...........A...........T...............C...........CG...T...G..TGTCA..AACA...CT.ACTAAACGTGA.CT...** 140

**ASLWS1CCTATGT-------------------------------------------------------------------------------TTTTAAACAGGTACCAGTTTCCTCCAATGAACCCGCTTTGGCACGGACTTTTGG** 201

**AYLWS1.......-------------------------------------------------------------------------------.........................G............................** 201

**HALWS1G..CCA------------------------------------------GTTTATTTATTTTGAAATCCAAAATATTTT------TTACAA.TGT.........................................C....** 225

**MbLWS1..GTAT.---------------------------GCATATCTTTGTTTTT---------------------------------------A.TT..................G.....T..A..............C....** 214

**MSLWS1...TCT.CAATTTCCGTCCTTTTAAGTTT---ATGTGTACGTTTGTTTTTTTCTCCTCGAAGTCTTTATGAACATTGAAAAATATGCACA.TT..................G.......................C....** 274

**SELWS1TT.CA.CAGGCATTAGGTGTTCGAACAATCGGTCGTGATGTGTGTATTGTCTTTGAACTACAGCTCTAACTTTATTCTAAAATATT.CA.T..T.................T...........A...........C....** 280

**SLLWS1TT.CA---------------------------------------------------------------ACTGTATTCTAAA-TATT.CA....T.................T...........A...........C....** 216

**ASLWS1GATTCACTATTGGCTGTCTTGGATTCATCTCTATCTCTGGCAACGGAATGGTCATCTATATATTTATGTCAACGAAGGTCTGTTCTATATACCCGTCTGCTTCAGGAGGGTTATGCTCTTTATTTTTGTTTTGGCTAATT** 341

**AYLWS1..........C........................A............................C................C.C....T....T.A........................C...A........TT.....** 341

**HALWS1..........C........C........T.....T.......................C..T..C..............-------.GG..TAT------..ATCCCTTT..TAT..GCA.CA.C.AA.C.GTTGAT...** 352

**MbLWS1..........C........................A...................T..C....................-------.....AA------AC.GTAATTTC..G.TTAAA..GC..C...A..CTTA..AA** 341

**MSLWS1.......A..C.......C................A.........................T.................-------.C...AATA..CTA.C..CATT.AC.G.TT.GAA.....C..C...C--A..CC** 405

**SELWS1.......A..C...GT......T........C..TA.....................................A.....-------.AT.TTA.------..ACCT.CAT..G.TTAG.CAGC...----.AT.AAG.AA** 403

**SLLWS1.......A..C...GT......T..T........TA.....................................A.....-------.A...ATT------AG.TAA.CAT..T.TTAG..AG..C.----.GT.AG..AA** 339

**ASLWS1TTTGTTTATGCTTAACTTACTTTTATAATATTTCTATTTCAGAGTCTTAAAACTCCATCAAACTTGCTAGTTGTTAATCTCGCTTTTTCCGACTTCCTCATGATGTGCGCTATGTCTCCGGCTATGGTAGTAAATTGTTA** 481

**AYLWS1...T...TC...........G.....T-...........................................G.......................T..T.......................G.....G...........** 480

**HALWS1...T..--------...A.GCCC....T.TA..TCT..A....................C...........G..G.....T.....C...........G...........G........T...........T........** 484

**MbLWS1C..TGC--------.T...---.A.CT..T--.T.T..G.............................C..G..A...........C..T..T...................................G..C........** 468

**MSLWS1....AC--------.T.G.AA.CA..TG.T--.TCT................................C..G..A...........C.....................TG..................G..G........** 535

**SELWS1A.CTA.--------GACA....GA...T.T-.CTCT..AT.........................A..T..G..G.....T.....C.....................TG..................T..C........** 534

**SLLWS1A.CAG.--------GACC.T..GA..GT.T-.CTGT..AT.........................A..T..G..G.....T.....C...........T.........TG..................G..T........** 470

**ASLWS1CAACGAAACATGGGTTTGGGGTGAGTA-----------TCTCAGCAAAT-------------------------------------------------------------------------------------------** 519

**AYLWS1...............A...........-----------..ATGC....--------------------------------------------------------------------------------------------** 517

**HALWS1...............A......A....TTAAGCAGATA.TAT..T..TATATTCCTTACTAATTCTAGGTACACTTAAATACGAAAGTAGGTAACTCAGTCAGGCTTTACGCCAAAGCTACTGAACCTATAGAAATTTTT** 624

**MbLWS1T..T..................A...T-------GTTT...TCAA.C---------------------------------------------------------------------------------------------** 508

**MSLWS1T.....................A...T-------GATA..ATC.T.CG--------------------------------------------------------------------------------------------** 576

**SELWS1T..............A......A....--------A-GCTA..AT.TT--------------------------------------------------------------------------------------------** 573

**SLLWS1T........G.....A......A....--------AAGCTAG....TT--------------------------------------------------------------------------------------------** 510

**ASLWS1----------------------------------------TAGTATTGTTACCATGATAATAT-------------------------------------------------TATTCTTTTTTA----------------** 554

**AYLWS1------------------------------------------......--------G......-------------------------------------------------.C.--...G...----------------** 540

**HALWS1GTTGCACGGGTAATCTAGAGCCTAGGAAAGGACTACTAAA...GC.AC..TTT..CCAG..GCTTAAAGAATCCCCCTTGAGACGCGGACGGAACCGCGGGGAAAAGCCAGT.G.GTA.AAAG.CGCTAAGTATTTTGTG** 764

**MbLWS1-----------------------------------------------------.A.CA...T.-------------------------------------------------.GC.T..G...---------------TA** 531

**MSLWS1----------------------------------------.T.ATGACA.TG..AAT....GC-------------------------------------------------....TC.ACA..--------------TA** 613

**SELWS1----------------------------------------.G.AC.G.A.TT.TCA.ATGAG--------------------------------------------------.G.GT..GA.G.--------AATACTTA** 615

**SLLWS1----------------------------------------.G.AC.CT..TTTT.CCA..C.--------------------------------------------------..AGTG.AAACG--------ATAAAATA** 552

**ASLWS1-CATTCTGTTACCACTATTATTGGCGTTCCCTCAGGTCCTCTTGCATGTGAACTCTACGCTTGTGCGGGCTCGCTATTTGGATGTGCATCGATTTGGACCATGACAATGATAGCTTTCGATCGCTACAATGTCATTGTGA** 693

**AYLWS1-T.....A....GG..G.A...T.T....................G.......................T..C...........................................................T..C....** 679

**HALWS1TTT..T....T.T.A.T..GG.T.TC....T.T.........C..T........T........C..T.....T........T.....C...........G....................C..............C..A.** 904

**MbLWS1TT...T.C.A......C..C..C.TT...TT..............T...........T..............T..C........................................................A..C....** 671

**MSLWS1TT.C.TAC.A..T..CC..C..AATT.CT.A...........A.......................T..........................................G....C.....C...........A..C....** 753

**SELWS1TGTCGT.C.C.T..TAC..C.CCA.A..GTT.T............G........A.....G.....C..A..T.....C.....C.....A..............T..............C.....T.....A..C....** 755

**SLLWS1TTT..T.C.C.T.G.ACA.T..CATA.CT.A.T............G........A.....A.....T..G..T........G........T..............T.....T........C...........A..C....** 692

**ASLWS1AAGGTATCGCAGCCAAACCCATGACCAACAACGGAGCACTTCTGCGTATACTCGGCATTTGGGTGTTCTCACTCGCGTGGACTCTCGCACCATTCTTCGGCTGGAACCGGTAAGGACTCCAAAACTTAATTTTCGT---A** 830

**AYLWS1..........G....................................................C............T....................................A...C....C....T....AT..T-A.** 818

**HALWS1..........C.....G..A...........T..G..T.....A.....T........C...T..........T..A........T..T..T..............T......ATGT.TAT------GG.G.C.T.GTG.** 1038

**MbLWS1..........C.....G....................T...........T............T..........T..A...........G...........................--.TCCTGAG.T.A...TACGTTG** 809

**MSLWS1..........C.....G..............T..G..T........C...........C..............T..A........T..G.................T......A.GA..T.CC.A..TG....TACGTCG** 893

**SELWS1.......A..C.....G....................T........C..C..T..A..C....CT.....G..T..A.....C.....G..T......................AT.AGATC.GGAA..GCACTT.TTT.** 895

**SLLWS1.......A..C........A........T........T.....A..C..C........C....CA........T..A.....C.....G..T......................AT..GGTCG.AAA....A.GACTGG.** 832

**ASLWS1ATAAACAA--------------------------------------TAAACAAGGT------------------------------------------------------------------------------------** 848

**AYLWS1........--------------------------------------.C..T....C------------------------------------------------------------------------------------** 836

**HALWS1.A.TG.TT------------------------------AAGTTCCACTG.T.....------------------------------------------------------------------------------------** 1064

**MbLWS1...GTTT-------------------------------------CAC...AGTA.------------------------------TAAAATTAT-------------------------------------------GTA** 839

**MSLWS1...GTGT.AAGGTGCTATTCGAACCTCTGGTGGTGCGGCAGTTGCACG..GC.A.CCGCGGATACACGACTGGTTTGTATTCCTATAAAACCATTCCGAACGCCACCACATGTGCTGCCCGCTGCCGAGTTTCAATAGCA** 1033

**SELWS1...TT.GT------------------------------TAGTATCTCT..TTTAA.------------------------------------------------------------------------------------** 921

**SLLWS1G..CTGT.------------------------------CTTTATAG.G..TC.AA.------------------------------------------------------------------------------------** 858

**ASLWS1------------------CATATTTAACGTATTTCAATACA------ATATTTCAGTTATGTACCTGAAGGAAACATGACAGCGTGTGGCACTGACTACTTGTCCAAAGATTGGTTCAGCCGAAGCTATATCCTTATCTA** 964

**AYLWS1------------------.......................------.................................................................................C...........** 952

**HALWS1------------------ACGT....--TATA.G..T.T..------.....A...A.....G..G.....G........T.....C..T..C.....T.............................C.....A.....** 1178

**MbLWS1CCT---------------ACCTAC...TT.TACGT.T.------ACT.....A...A.....G..C..............T..A......................................C.....C...........** 958

**MSLWS1CCTTTACAAAAAACAATAA..TG..G..CCTAC.A.T.TT.AGCATT..T..A...A.....G..C..............T..T........A..............G..............C.....C.....G.....** 1173

**SELWS1------------------A.A.CA...TT.C.....TCC..------...C.G...A..............C........T..T.................A.......................T..C.....G.....** 1037

**SLLWS1------------------TT..AG...TT.G..G..TCTT.------...C.G...A........G.....C........T..T.......................G.................T........G.....** 974

**ASLWS1CTCCGTATTCTGCTACTTCATGCCTCTTCTGCTTATCATTTACTCTTATTTCTTTATTGTCCAGGTAATGCAGAAAAACCTTTAGGTCTAATGAACTATCTTGGTTATTTTTTTGTAAATAACGGTGAATTGTAATG---** 1101

**AYLWS1......T...GTA.....TC............................C..............................A......A......G----.....T.--...A..........................---** 1083

**HALWS1............T....................G.....A........C.....C............CCATTTG....GAA..CCTAA.CTCAGCAAT.T.AATGATCC.C.GA..G.....TAC.T...CAAT.CC-TA** 1317

**MbLWS1......T...GTT......T...................A..T.....C....................T.......TA.A..TTAGA.TCATG.AGCATC.TT..CCG.AACA....G.C.GAAGC.CACTGC-CCGAC** 1097

**MSLWS1......C...GTA..........................C..T..C..C...........................-TAGA..T.AGTCT.CTG.AAGATC.TTA.TC.AAACACA.TG.CTG.CAT.T.AAGT..CATA** 1312

**SELWS1T.....C...GTG..T..............C..C.....C..............C.....A......--------...TAGGC.CTA..CC-T.CTATA.ACAC.GT...A...C-.GTACCTTT.T.GAC...GC----** 1163

**SLLWS1......C...GTG..T...T.......C..C..C.....C..............C.....A....C.--------.GTA..A.GCAA..GTATTTTATAT.A.TAGG.ACC..CC...TATTTTC.TCG.A..G..----** 1102

**ASLWS1-------------TTTCTCTATTGCATTCAGGCCGTAGCAGCTCACGAGAAGGGAATGAGGGAACAAGCTAAGAAAATGAACGTAGCTTCCCTCAGGTCTTCAGAAGCGGCAAATACCAGCGCGGAGTGCAAATTGGCAA** 1228

**AYLWS1-------------........C.TT...................................................................................................................** 1210

**HALWS1AGAATATTATACCAA.T.A.T...T.............................G...................................T..G.....G..G.....A..T..C..A.....A................** 1457

**MbLWS1TGGAATAT-TAATG..T.TCT......C.......................A.........................................T.................T..C........T................** 1236

**MSLWS1TAATATATATCAT.C.T.TCTC..T..........................................................................A........A..T..C........T................** 1452

**SELWS1AACTTTACGATTC--.T.TA...TA........T..............A...................................G.....T..A........T...........C..A.....T..............T.** 1301

**SLLWS1AGGAAC-CAACTCAACT.T.C..T.........T..............A...................................G.....T..A........T..G........C..A.....C................** 1241

**ASLWS1AGGTCGAACTCATTCTAACCTGCGTTAATGCTTTGTCT------CGGTATTCATAGAAATTCTATTAACCGCAACATAATATTTCAGGTAGCTCTAATGACCATATCACTCTGGTTCATGGCGTGGACACCATACCTCGT** 1362

**AYLWS1....T.............GT.A....GC.A..------------...A............C.......T...........G..........................T..A.............................** 1338

**HALWS1....A..GTCTCG.AATGATCAAT...TACAC..T.ACCCAATTTA-C.CCT..TCTTG.AT.T.A..TT..T.TT..C.-...........GT..........T.....G..........................T..** 1595

**MbLWS1........A......A..TTAAATA.GT.TACC.AG.CTC---A.AAG.....C.A....C.....T.TTA....C..T.G..A....................T..G..G...........A........G.....S..** 1373

**MSLWS1....AA..TA.G....C.TTAAGTAC.CCT..CA.GA.-----ATAAA..CA..CCT....-..C...TG....TC..TCG..A........GT..........T.....G.................T..G........** 1586

**SELWS1....TTG.TA....TCTTTGGAGAA....TAACAACAACTTTTAT.TC.G.TTCGA.TTC.T.T..C..G.TT.A.C.T.G..A.......................T..G....................C.....A..** 1441

**SLLWS1....TTGC.A....AA---G.ATT.CTTC.AG..A.G.TTCTTATAAC.CCTT..T.TTC.G.T..C.TGATT.A.C.T.G..A.....G...........T..T..T..G....................C.....A..** 1378

**ASLWS1GATCAACTACACTGGTGTGTTCGAAAGTGCGCCCATCAGCCCCCTTGCTACCATCTGGGGCTCACTCTTTGCTAAGGCTAACGCTGTCTACAATCCTATAGTATATGGCATCAGGTCTGTATTTGGCCATTATTTGTTTT** 1502

**AYLWS1............G........T.............................................................................T.....C...................AA....T..A.GG..** 1478

**HALWS1...A........G........T.....C........T.....T..G.....T....................C.....C.....C..T...........T................T...G...TCGTTCA.A-------** 1728

**MbLWS1...T........G.....A........C.....G........T........T.................C..A.......................C..T...........T....T...T...CTTTGACGAAAA----** 1509

**MSLWS1..................T........C..A...........T..C.....T...............................................T................G...T....TATGA.GAA.A----** 1722

**SELWS1...T......G.G.....C........C..AA.......T..T.....G.....T........T...................................T.....C...............A..TT.TTC..AAATA.AC** 1581

**SLLWS1...T........A.....C........C..A........T..T.....G..............T........C..A.......................C.....C............C.....TCTTGC.CAA.TA-AC** 1517

**ASLWS1TTTTAAACGACTTCAAAAGAAAAGTAGGTTCTCAGTTCGACCTTTATGTATGACTGTATGTTTGTACGCGATTATCTCGCGTTTGACTGAACGGATTTCCATGTAGTTTTCAGGATATAGATATACTAAAACCTTCTCCA** 1642

**AYLWS1CCG..GC.A.A.GGC..GA.GGG.A.CCC.TAT..A.T---.G.C....C..T....C...C...C..TC-CG..G..A.AGCC-..CA...TC..ACGTG..GGA...C-----...G....GG---------....T.** 1599

**HALWS1-----------..AT----TTGT.AG.CACTCA.A.ATA.AA-AAT...TAT.AA..GACA.AAA.TAAT.AA.ATATCGT..C--------------------------------------------------------** 1796

**MbLWS1-----------.CAT---------A..C..T.T.AAATACT.AAAGCAGC...AC.G.AC..CAA.T..A.AAT.A.T.TT.CC--------------------------------------------------------** 1573

**MSLWS1-----------.CAT---------A.AC.ATCAGTAGG--TGACCGCACC..TA---.GT..CTA....A.AATG..T.TTG.---------------------------------------------------------** 1780

**SELWS1AAAAT..GT..C.AT-.CT.C.CTA.AAA.TCAGT.A.C.AA-GA..A..CCTA...GAC..AAA..TGTTA.--GAATGT.AC--------------------------------------------------------** 1661

**SLLWS1ACAAC.-----..AT---ATC.TT.T.TC.ACA.TAA.T.AA.AGG.A-----A..C.AC..AAA.TTG.TA...GAATGT.AC--------------------------------------------------------** 1588

**ASLWS1GAATGT-AATGAAAACCGAACGTGACGTTGCCCACGTGGAATCGTGGATCCGTTTTTGCTTTCTTGGATTGTAGATTTTCAGAGACGATCGCTAAAAAAAGAGAACCGACGGCGATTTCAAAACCAAACATAAAAAACTG** 1781

**AYLWS1--..C.-.C.A.T...---------------------------------------..TT...TC.AA.C..A.C.G...GC.C..---------------....--------.AC..C....GTG---------------** 1659

**HALWS1--------------------------------------------------------------------------------------------------------------------------------------------** 1796

**MbLWS1--------------------------------------------------------------------------------------------------------------------------------------------** 1573

**MSLWS1--------------------------------------------------------------------------------------------------------------------------------------------** 1780

**SELWS1--------------------------------------------------------------------------------------------------------------------------------------------** 1661

**SLLWS1--------------------------------------------------------------------------------------------------------------------------------------------** 1588

**ASLWS1CCGGGTGTAGAATCAATCCCATCCCTTCCCTTTTAATAATAGACGTTCAAAAGTGCCTCTCCTATTATAGCAAAATCGCATTTTGCTCACTGATTTCAGGCAGCAAAACCAGTATGTTTGAGCTACTGAAGTGAAAAAGT** 1921

**AYLWS1------...A...GTG....----.AC...C.G...----------------------..T...AA...AA.G...GAT.-----------A.AC.A.ATAG.T....A..A-..A.A...CG....------------.** 1743

**HALWS1--------------------------------------------------------------------------------------------------------------------------------------------** 1796

**MbLWS1--------------------------------------------------------------------------------------------------------------------------------------------** 1573

**MSLWS1--------------------------------------------------------------------------------------------------------------------------------------------** 1780

**SELWS1--------------------------------------------------------------------------------------------------------------------------------------------** 1661

**SLLWS1--------------------------------------------------------------------------------------------------------------------------------------------** 1588

**ASLWS1TATTTTGTGTTTGGTTTGAAGACTGTTCCCTTATTTCAAAAATATTTTTTTTTTTAAGGTCGTCAAATGTTTGAATATCGTACTTAGCTGCGAGATTATGGTAGATAGGCAAACGACAATTGAACGTATTCTGACGCCAA** 2061

**AYLWS1..CCA.CCA------------------.A...CCAG.G.....-----.GG...G..C.AAA.G.GG.AA..CGT.G.T...---------.G....G.A..T...------------------.CC.....A..A....** 1833

**HALWS1--------------------------------------------------------------------------------------------------------------------------------------------** 1796

**MbLWS1--------------------------------------------------------------------------------------------------------------------------------------------** 1573

**MSLWS1--------------------------------------------------------------------------------------------------------------------------------------------** 1780

**SELWS1--------------------------------------------------------------------------------------------------------------------------------------------** 1661

**SLLWS1--------------------------------------------------------------------------------------------------------------------------------------------** 1588

**ASLWS1ACTATTGTTGTAGCCACCCGAAGTACCGCGCTGCTCTGTACCAGAGATTCCCGTCCCTGTCGTGTCAGGC** 2131

**AYLWS1.T.......................T......................................C.....** 1903

**HALWS1-----------.......................GT................A..A........C.....** 1855

**MbLWS1-----------..T..............T................AG..T.....G........C.....** 1632

**MSLWS1-----------...................................G........G........C.....** 1839

**SELWS1-----------................................A.AG.....A..G..A.....C.....** 1720

**SLLWS1-----------..................................AG........A..A.....C.....** 1647

**(a)**

**ASLW2ATGGTCATCTACATATTTTCA--ACCACCAAGAGCCTAAAGACACCATCAAACCTGCTGGTAGTGAATCTAGCTTTCTCCGACTTCCTCATGATGTGTGCTATGGCTCCAGCTATGGTTATTAATTGCTACAACGAAACA** 138

**AYLW2.....................--T.............G................................C.................G...................................................** 138

**HALW2.................C...--T.............G........G..C.....T...........C..C..A..............T........C...........G.................T.....T.....G** 138

**MBLW2.................C...--..............G...........C.................C..C.....T...........G...G....C...........G.........G.............T......** 138

**MSLW2.....................TG...G......A...G.....G..G..C.................C..C.................G......GTC...........G........C.....................** 140

**SELW2.....................--..............G...........C.................C..C.....T...........G........C...........G.........G.............T......** 138

**SLLW2.................C...--..G..T..A...T.G...........G...T.A..T.....C..C..C.................G........C..G.....C..G........A..A.....T............** 138

**ASLW2TGGGTATTTGGTCCGTTTGCTTGTGAACTTTATGGTTGCGCTGGCTCACTATTTGGATGCGCATCTATATGGACCATGACGATGATCGCCTTCGACCGCTACAACGTCATCGTGAAGGGTATTGCCGCCAAGCCAATGAC** 278

**AYLW2................................C................................G..............C.............................T...........C...........C.....** 278

**HALW2.....G........A.....G........C..C..A.....A.....G....................T...........C.....T..A..........................A.....C.....T...........** 278

**MBLW2....................C..C.....A..C.....T.....A.................C.....C.....A.....C.........................................C.....T..A........** 278

**MSLW2....................C..C.....A..C...........................A.......C...........C..............T..........................C.....T..A..C.....** 280

**SELW2....................C..C.....A..C.............................C.....C.....A.....C.........................................C.....T..A........** 278

**SLLW2.....G..............C........C..C..C............T..........T........T...........C.....T..T..............T.................C...........T.....** 278

**ASLW2TAAGACTGGAGCTCTTCAGCGCATACTCGGAATC** 312

**AYLW2....G......................T......** 312

**HALW2C..AT.C........A..................** 312

**MBLW2............C..............T......** 312

**MSLW2C..........................T......** 314

**SELW2C...........C..............T......** 312

**SLLW2C...T..........A..................** 312

**(b)**

ATGGCAATTGCCAGCTTGGACCCCGGCCCAGGAGTCGCCGCGCTGCAGGCGTGGGGCGGGCAGGTGGCCGCCTATGGCGCCGCCAACCAGACCGTCGTCGATAAAGTGCCTCCCGACATGCTGCATATGGTCGATCCATACTGGTAAGTATAACAGACACCAAATTAAGGCGCGTATTCTGAAACAACTCCTAAATCTTGAAGGATTGGTCACAATACCAATTCGAGGAAGCAATAAACATACTTGAGTATTGACTTTGACCTAAAATGACTGTCTCATTCTAGGTATCAGTTTCCACCTATGAACCCGCTATGGCACGGTCTTTTGGGTTTCACTATCGGCGTTCTCGGCTTCATCTCAATCACTGGCAATGGAATGGTCATCTACATCTTTACTTGCACCAAGGTACATATGTACATATTATAACCACAGTGGAAATATCTAGGTGGTATAATAAATTGCACTGCTGTTTGCTGAAGTTTAATAAGTGCTTCATTTTCAGAGTCTTAAAACGCCGTCAAATCTACTGGTCGTCAATCTTGCTTTCTCCGATTTTCTGATGATGTGCGCGATGTCTCCGGCTATGGTTGTGAACTGTTATAATGAGACTTGGGTATGGGGTAAGTAATTGTTTAGCTACAAAGCTAAGTTGCCCAAAAAGATATAATTCTAAGTTTTCAAGTGGTTTAATTTTGGTTTTCGTTTCAGGTCCTCTGGCTTGCGAACTGTACGCCTGCGCGGGTTCTCTATTTGGCTGTGCTTCAATCTGGACAATGACTATGATCGCCTTCGACCGCTACAACGTCATCGTGAAAGGTATCGCCGCCAAGCCTATGACCAACAACGGAGCTCTACTGCGCATCCTTGGAATCTGGCTGTTCTCTCTTGCATGGACGCTCGCTCCATTCTTCGGCTGGAATAGGTAAACGACTACCTAATAATGTCAACAAACCTCTATGTTTCCTGCTCATCACTGTGATGACCAAATGGTTTAAGAAAATTAACAATGATTATGCTTCTGTTATTCCAGATATGTCCCAGAAGGTAACATGACTGCTTGTGGAACAGATTACTTGAACAAGGACTGGTTTAGCCGAAGCTACATTCTCATCTACTCCGTCTTCTGCTACTTCATGCCTCTTTTGCTTATTATCTATTCTTATTTCTTCATTGTACAGGTAAGAATATTCTTTCTTCCCACGACATGCAAAATTAACTCAACTGTGTTGTFTAGTTAATTTCTTAATAATGCCTCTTAATTTCAGGCTGTAGCTGCCCACGAGAAAGGCATGCGTGAACAAGCCAAGAAAATGAATGTGGCTTCCCTTAGGTCTTCCGAAGCGGCCAACACCAGCGCTGAATGCAAACTAGCCAAGGTAATGCACGAGCACTAAAGTCCAAAACAATAAATTTCTGATCTGTTGTTGTTTTCGTCTCTTTTTATTTTATATTATCTAATCTGTAATATTTGTAGGTTGCGTTGATGACCATCTCTCTGTGGTTCATGGCCTGGACACCGTACTTGGTGATCAACTACACAGGAGTATTCGAGAGCGCCCCCATCAGCCCTCTGGCTACCATCTGGGGCTCCCTGTTCGCCAAAGCTAACGCCGTATACAATCCTATTGTATATGGTATCAGGTCCGTAACTATATTTGACAGCTTCTGAAGTACCACCAATCTGTATCTATACAAAGAAATGTTTTTCATTTGCTTTGATCCTGATTTCTCCAATATGTTCAAAATTAGTGCCAGCGATTTTTATGCAACTAAACGGGACTATTCTTTCTTTTCCGCCACTGTTGTAACTCCTGTAGCCAGGATCTCCAATGAATCCCAACTTCTGGGGCTTCTACACCACTAGATAGAGTCGATTTATGATTTAATCGAATGTTTGTGTTTTCAGCCATCCGAAATACCGTGCTGAGCTATACAAGAAGTTCCCATCGCTGTCGTGCCAGGCGTCCCCCGACGAGAGCGGCTCGGTCGCCTCCGGCGCCACCGCCACCTCCGATGAGAAACCCGCTGC

**(c)**

ATGGCTACAGGACCAGGAGTCGCAGCTCTTCAAGCATGGGGCGGGCAGGTTGCCGCTTTCGGCACCGCCAACCAGACCGTCGTCGACAAGGTGGCACCAGAAATGCTACACATGGTCGACCCTCACTGGTAAGCGCATTGTTCCAATCTTTACACCAATCACTATTCATGAATCATCATAATTGTGATAATTATCGAATTAAAAGCTGATCCAGAACATGCAATGTAATCATTATAAAGCCTATTTTTCCAAAATGAGAAATCACAAAAACACTTTGAATTATAATGAAAATGTTACTGTACAACTTTTACTTATTACTCACACGATCTTATTTATTTTATCTAGGTATCAGTTCCCTCCTATGAACCCCCTTTGGCACGGACTTCTGGGCTTCACCATTGGAGTCCTAGGTCTCATCTCAATCATTGGCAACGGAATGGTCATCTACATCTTCTCTTCAACCAAGGTTTGCCTTTAAGTTTTTAGAAATATTGCCTGGGTTTTAAAGTTTAAACTTATTGCATTTCATCGATTACGTTTCCTTGCAGAATCTGAAGACCCCCTCTAACCTGCTGGTGGTCAATCTCGCCTTCTCCGATTTCTTGATGATGTGCGCGATGGCTCCAGCTATGGTTATCAACTGCTACAACGAAACATGGGTATTTGGTAAGTGTAAATTTTCTCAATCTCTTCTTTTCAGGCAAAAGAAGTGTAAATTTTCTCAATCTCAATTAAAAGAAATTTCACATCATTTCATTGGATTTTTCTTCAAATTTGTAATGAATTGTCTAATATCTCTTGTCTATAGGTCCATTCGCTTGTGAGCTCTACGGCTGTGCGGGCTCTCTATTCGGCTGTGCTTCTATCTGGTCTATGACCATGATTGCCTTCGACCGGTACAACGTCATCGTCAAGGGTATCGCTGCCCAGCCCATGACCAAGAACGGCGCTCTATCTCGCATCCTCTTCATCTGGGCCTTCTCTCTCGCGTGGACTCTTGCTCCTTTCTTCGGGTGGAACAGGTTTGTAATTGAGAACAAAAACTCGTTAATTAAATATTATAGTCATCAAAGTGTCAATATCTACCTATCAATCAATTAAAACATCATTTTACCTAACAGGTATGTGCCCGAAGGCAACATGACCGCCTGCGGAACTGACTACCTGACCAAGGACTGGCTCAGCCGCAGCTACATCCTGGTCTACTCAGTCTTCGTGTACTTTCTGCCTCTGCTCCTCATCATCTACTCTTACTACTTCATCGTTCAGGTAAATTATCGAACTCATTTCTTCTTCGATCCATAAATCTTGATCAAAGATCACAGCAAATTTTAGGATCTTGCACTGACACGTTGGATGGGTTTCAGGCTGTTGCTGCCCACGAAAAGGCTATGCGTGAACAAGCCAAGAAGATGAACGTTGCTTCCCTCAGGTCCTCTGAAGCCGCTAATACCAGCGCTGAGTGCAAACTTGCCAAGGTT

**(d)**

**Figure S2** **The genomic sequence of LWS opsins in seven noctuid species.** (a) LWS1 opsins showed seven introns. The red letters showed the homology region of primers for amplifying partial sequence of LWS2 using DNA as templete. The introns are shaded with grey. The seven transmembrane domains are shaded with yellow, in which the last two domains are separated by introns. (b)The fragments of LWS2 opsins from seven noctuid species showed no introns in the region. “.” = identical nucleotides; “-” = absence of nucleotides. AS=*Agrotis segetum*, AY=*Agrotis ypsilon*, HA=*Helicoverpa armigera*, MB=*Mamestra brassicae*, MS=*Mythimna separata*, SELWS1=*Spodoptera exigua*, SL=*Spodoptera litura*. (c) The genomic sequence of LWS1 in *O. nubilalis*. (d) The genomic sequence of LWS2 in *O. nubilalis*. The exons were showed using black letters and the introns were showed using red letters.


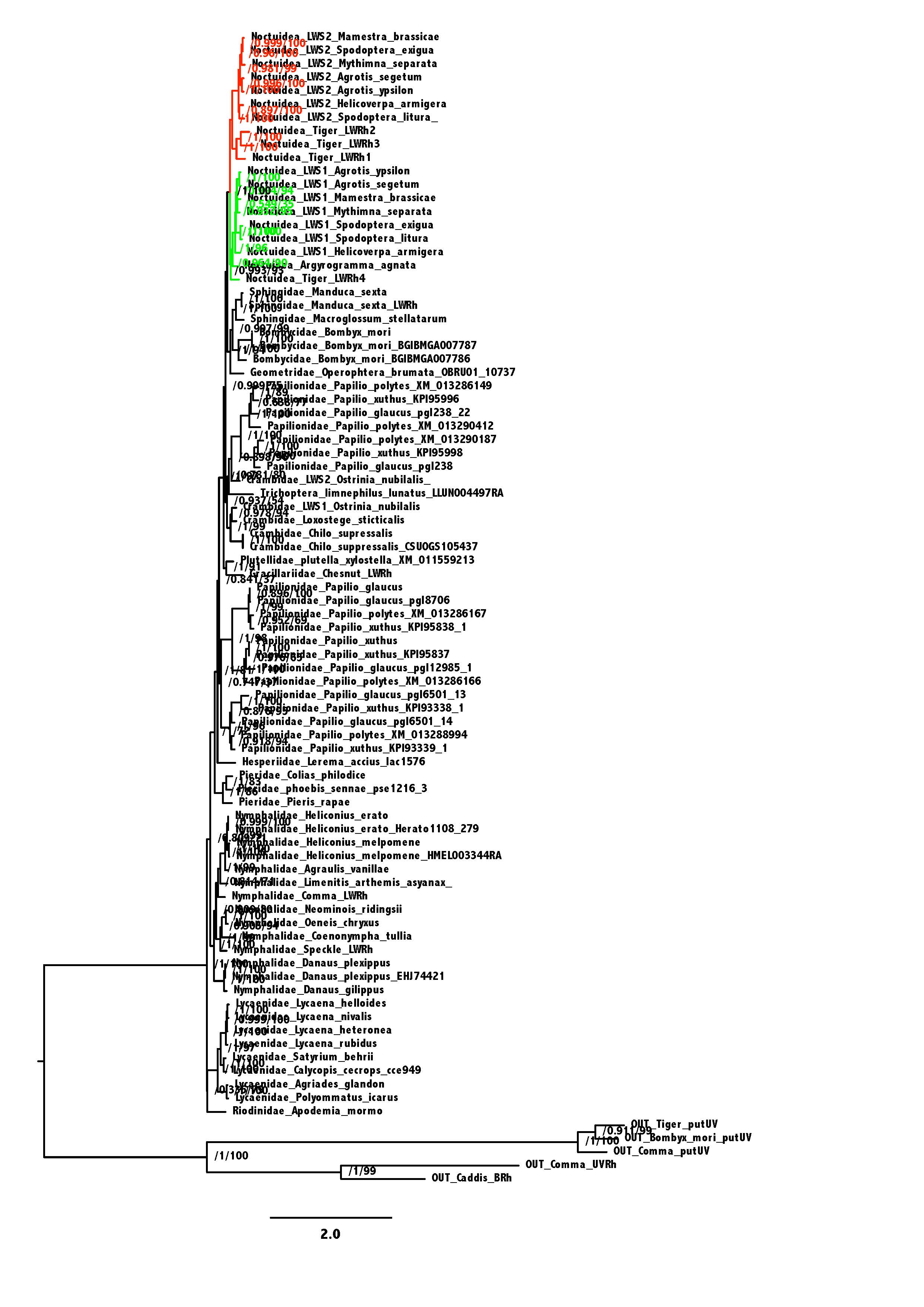


**Figure S3** **Maximum Likelihood tree with outgroup.**


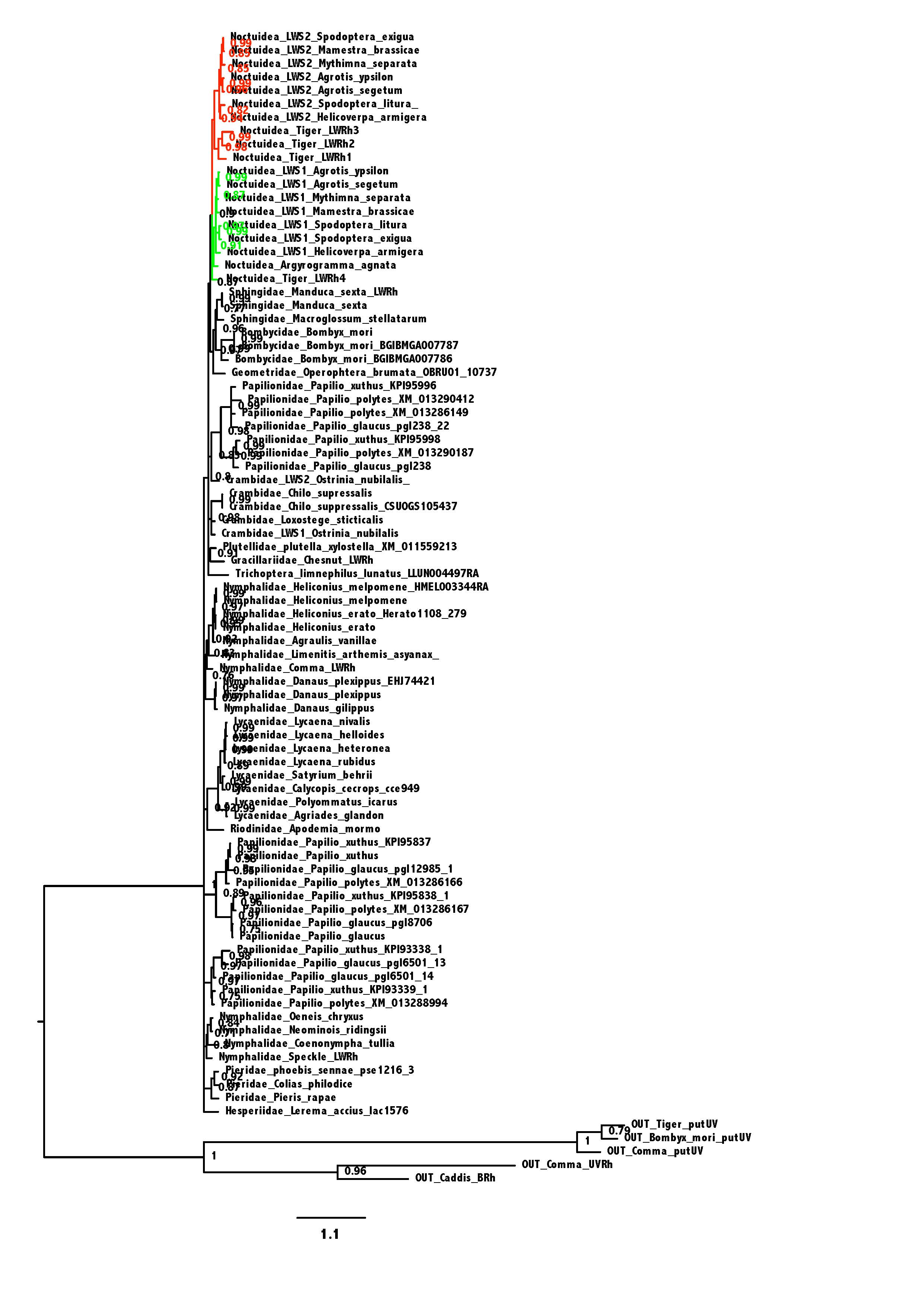


**Figure S4** **Bayesian tree with outgroup.**


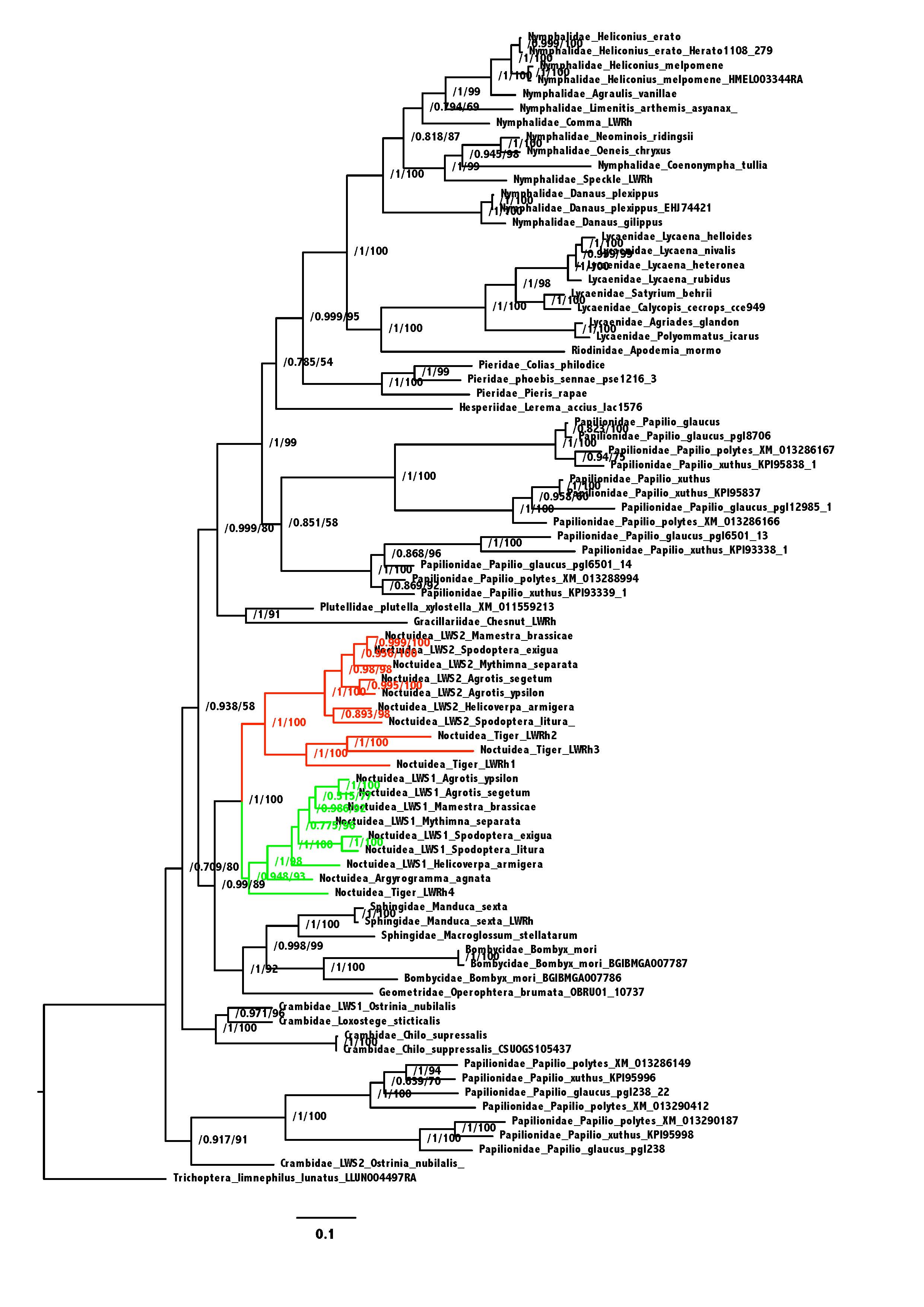


**Figure S5** **Maximum Likelihood tree with outgroup.**


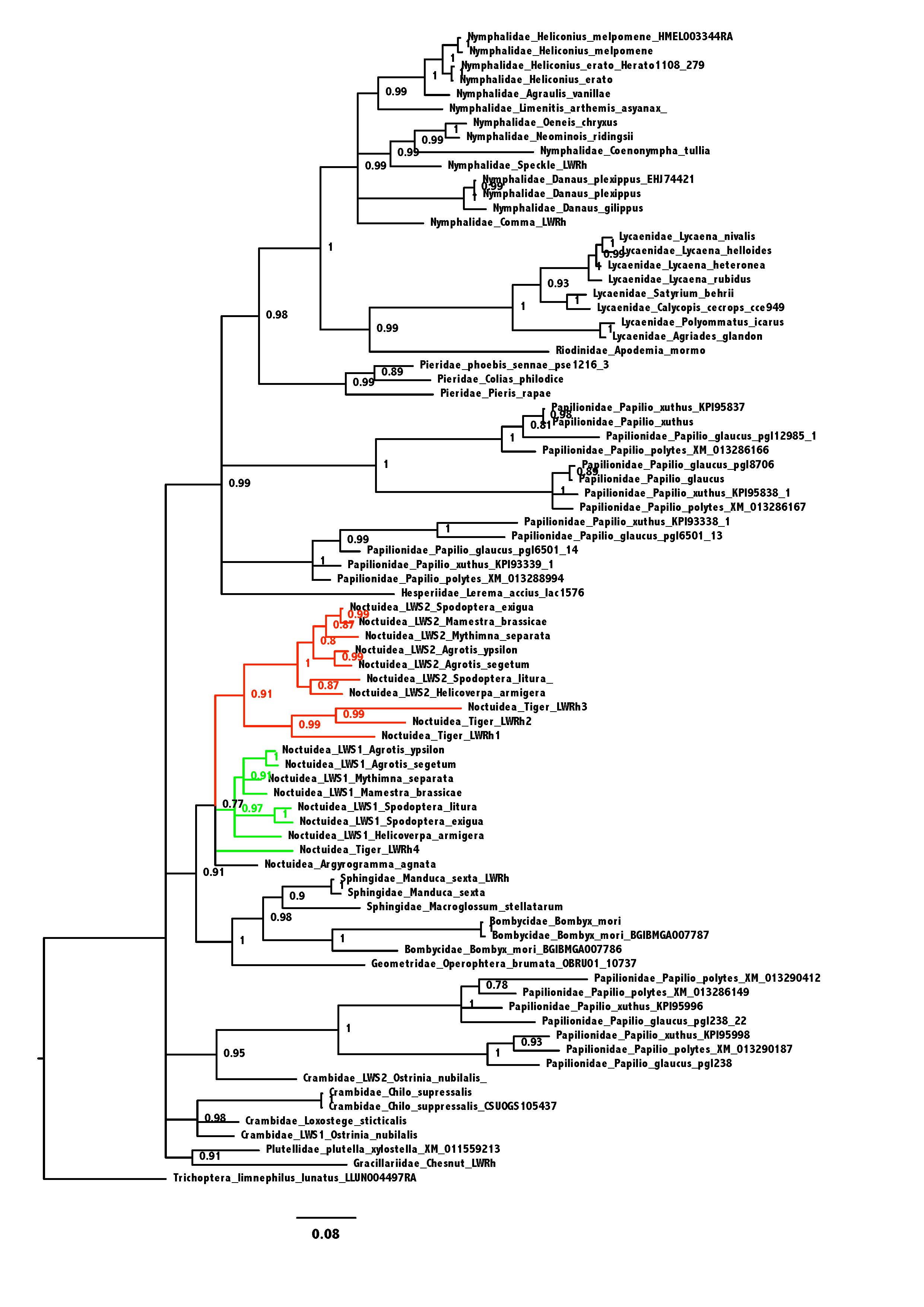


**Figure S6** **Bayesian tree with outgroup.**
